# Cordycepin, lactoferrin, and Sargassum fusiforme polysaccharides protects against RSV via M2-like macrophage polarization

**DOI:** 10.3389/fimmu.2025.1576069

**Published:** 2025-06-16

**Authors:** Xiaozong Fu, Panwen Huang, Yuting Zhang, Yanchang Li, Shichang Hu

**Affiliations:** ^1^ Preventive Health Care Department, Dongguan People’s Hospital Xiegang Branch, Dongguan, Guangdong, China; ^2^ Department of Respiratory and Critical Care Medicine, Dongguan People’s Hospital, Dongguan, Guangdong, China; ^3^ Hospital Infection Control Department, Dongguan People’s Hospital Xiegang Branch, Dongguan, Guangdong, China; ^4^ Department of Respiratory and Critical Care Medicine, Dongguan People’s Hospital Xiegang Branch, Dongguan, Guangdong, China

**Keywords:** respiratory syncytial virus (RSV), macrophage polarization, cordycepin, lactoferrin, Sargassum fusiforme polysaccharide, lung pathology

## Abstract

**Background:**

Respiratory syncytial virus (RSV) is a leading cause of respiratory tract infections, particularly in infants and the older population, with limited effective treatments available. Cordycepin, lactoferrin, and Sargassum fusiforme polysaccharides (CLS) are natural compounds with antiviral and immunomodulatory properties. This study investigates the therapeutic potential of CLS in RSV infection.

**Methods:**

A murine model of RSV infection was used to evaluate the effects of CLS. Lung pathology was assessed by lung index, histology, and bronchoalveolar lavage fluid (BALF) albumin and LDH levels. Viral load was measured by RSV mRNA and protein expression. Alveolar macrophage depletion was achieved using clodronate liposomes, and macrophage polarization was analyzed via flow cytometry and RT-qPCR.

**Results:**

CLS treatment improved lung pathology, reduced BALF injury markers, and decreased viral load. The therapeutic effects of CLS were abrogated by macrophage depletion, indicating a reliance on alveolar macrophages. CLS promoted M2-like macrophage polarization, increasing M2 markers and reducing M1 markers. *In vitro*, CLS reduced RSV-induced apoptosis and enhanced macrophage proliferation.

**Conclusions:**

CLS protects against RSV-induced lung injury by promoting M2-like macrophage polarization and reducing viral load. These findings support CLS as a potential therapeutic for RSV infections.

## Introduction

Respiratory syncytial virus (RSV) is a leading cause of lower respiratory tract infections worldwide, particularly affecting infants, young children, and the older population (1, 2). RSV is associated with significant morbidity and mortality, accounting for approximately 3.2 million hospitalizations and over 100,000 deaths annually in children under five years of age (3, 4). In China, RSV poses a substantial public health burden, accounting for 57.21% of pediatric viral pneumonia hospitalizations, with infants under one year representing nearly 60% of cases (5). Seasonal epidemics peak in winter (December–February), contributing to 41.67% of annual hospitalizations, while summer months exhibit the lowest activity (5, 6). Severe RSV cases are associated with complications such as respiratory failure (6.95%) and myocardial injury (8.43%), highlighting the urgent need for effective interventions (5).

Despite the high global burden, effective treatments for RSV remain limited (7, 8). Recent advancements in RSV prevention include the approval of three vaccines (7). Two of these are subunit vaccines: GSK’s Arexvy and Pfizer’s Abrysvo, which target the RSV F protein and are approved for use in older adults and pregnant individuals (9). The third vaccine, mRESVIA (mRNA-1345) developed by Moderna, is an mRNA-based vaccine that has been approved for use in older adults and is currently under regulatory review for use in pregnant individuals (10). While these vaccines represent significant progress in prophylaxis, therapeutic options for active RSV infections remain limited, particularly for high-risk populations such as infants and immunocompromised patients.

Natural compounds have emerged as potential candidates for the treatment of viral infections due to their favorable safety profiles and diverse biological activities (11, 12). Cordycepin, a bioactive compound derived from *Cordyceps militaris* (L.) Fr., has demonstrated potent antiviral properties against viruses such as SARS-CoV-2, dengue virus and hepatitis C virus (13–15). Lactoferrin, a glycoprotein with immunomodulatory and antimicrobial properties, has demonstrated *in vitro* efficacy against RSV, although its *in vivo* success has been limited (16–18). Additionally, *Sargassum fusiforme* polysaccharides, extracted from marine algae *Sargassum fusiforme* (Harvey), possess immune-enhancing and anti-inflammatory effects (19, 20). However, the synergistic potential of combining these natural compounds has yet to be fully explored in the context of RSV infection.

Alveolar macrophages (AMs) play a pivotal role in the pulmonary immune response, acting as the first line of defense against respiratory pathogens (21). During RSV infection, AMs can polarize into either the M1 or M2 phenotype, with M1 macrophages promoting pro-inflammatory responses and M2 macrophages facilitating tissue repair and resolution of inflammation (22, 23). Dysregulation in macrophage polarization has been implicated in the exacerbation of lung pathology during RSV infection, making macrophage polarization a promising therapeutic target (24, 25). Previous studies suggest that natural compounds, including cordycepin, and *Sargassum fusiforme* polysaccharides, have the potential to modulate macrophage polarization, shifting the immune response toward an anti-inflammatory M2 phenotype (26, 27). This polarization may play a critical role in mitigating RSV-induced lung damage and improving viral clearance.

While cordycepin, lactoferrin, and *Sargassum fusiforme* polysaccharides exhibit individual antiviral and immunomodulatory effects, their synergistic potential in modulating macrophage polarization during RSV infection remains uninvestigated. This gap is critical to address, as dysregulated macrophage polarization exacerbates RSV-induced lung injury, and no current therapy effectively targets both viral replication and immune homeostasis.

In this study, we investigated the therapeutic potential of a novel combination of cordycepin, lactoferrin, and *Sargassum fusiforme* polysaccharides (CLS) in a mouse model of RSV infection. We hypothesize that CLS protects against RSV-induced lung pathology by promoting M2-like macrophage polarization. By exploring its effects on lung injury, viral load, and macrophage polarization, we aim to provide insight into the mechanisms underlying the protective effects of CLS and its potential as a therapeutic intervention for RSV infections.

## Materials and methods

### Reagents and antibodies

Reagents used were as follows: CLS (Cordycepin, Lactoferrin, *Sargassum fusiforme* polysaccharides, REGENERON, USA), ribavirin (R9644, Sigma-Aldrich, USA), Clodronate liposomes (40337ES08, Yeasen, China), human RSV strain A2 (Wuhan University Virus Institute, China). Antibodies: anti-RSV-F (ab94968, Abcam, UK), anti-CD11c (ab254183, Abcam, UK), anti-F4/80 (ab300421, Abcam, UK), anti-iNOS (ab49999, Abcam, UK), Goat Anti-Mouse IgG H&L (Alexa Fluor^®^ 488) (ab150113, Abcam, UK), Goat Anti-Mouse IgG H&L (APC) (ab130782, Abcam, UK), Goat Anti-Rabbit IgG H&L (Alexa Fluor^®^ 488) (ab150081, Abcam, UK), Goat Anti-Rabbit IgG H&L (APC) (ab130805, Abcam, UK), anti-F4/80-FITC (11-4801-82, ThermoFisher, USA), anti-iNOS-PE (12-5920-82, ThermoFisher, USA), anti-CD206-PE (MA5-16872, ThermoFisher, USA), anti-CD206 (18704-1-AP, Proteintech, USA). Other materials: SYBR Green PCR Master Mix (Applied Biosystems, USA), Dulbecco’s modified Eagle’s medium (DMEM) (Gibco, USA), and fetal bovine serum (FBS) (Gibco, USA), Mounting Medium with DAPI (ab104139, Abcam, UK).

### *In vivo* experiment

RSV-infected model was carried out as previously described (28). Six-week-old male BALB/c mice (SPF grade) were obtained from Guangdong Zhiyuan Biomedical Technology Co., Ltd. (Guangdong, China) and kept under pathogen-free housing conditions in a 12-h light and dark cycle. The mice were randomized into 4 groups (n = 3 per group): (i) control group; (ii) RSV-infected group; (iii) RSV-infected and ribavirin-treated group; (iv) RSV-infected and CLS-treated group. The mice received an intranasal infection of RSV (human RSV strain A2, Wuhan University Virus Institute, China). Intranasal infection was performed on awake mice by trained researchers: 50 µL of RSV suspension (10^6^ PFU in PBS) was pipetted into the nostrils (25 µL/nostril) without anesthesia to avoid confounding effects on respiratory dynamics. Control mice received an equal volume of PBS. In the ribavirin-treated group, an oral dose of 46 mg/kg ribavirin was given to achieve therapeutic plasma concentrations according to a previous study (28). In the CLS-treated group, mice were oral administrated with CLS compound, consisting of 100 mg/kg cordycepin, 10 mg/animal lactoferrin, 200 mg/kg *sargassum fusiforme* polysaccharides. At day 7 after infection, all mice were anesthetized with CO_2_. The lungs of the mice were excised and measured. The lung ratio was calculated by the following formula: lung ratio = lung weight/body weight. The left lungs were used to collect BALF and the right lungs were fixed and paraffin embedded.

Sample sizes (n=3 per group) were determined based on preliminary pilot studies and consistency with prior murine RSV infection models (29, 30), which demonstrated comparable effect sizes with similar n. While larger samples may enhance power, this study prioritized feasibility and reproducibility, with statistical significance confirmed via ANOVA and Dunnett’s tests (p < 0.05). The dose of cordycepin, lactoferrin and *sargassum fusiforme* polysaccharides were determined based on individual component efficacy from previous studies (31–33), with the combination optimized to maximize synergistic effects while ensuring safety (**Supplementary Figure S1**).

### *In vivo* depletion of alveolar macrophages

Alveolar macrophages were depleted as previously described (34). In brief, clodronate liposomes (Clip) at a concentration of 5 mg/mL (100 μL of suspension) was administered intranasally to mice 24 h before infection, and continued daily until the mice were executed for sample collection. When CLS was administered concurrently, there was a minimum interval of 6 h between the Clip and CLS doses to avoid potential interactions between liposomal delivery and oral absorption.

### Histologic analysis

Sections of lung tissue (4 μm) were obtained from paraffin-embedded samples, underwent a series of dewaxing and hydration processes, and were subsequently stained with hematoxylin and eosin (Beyotime, China).

### Immunofluorescence analysis

Slices (4 μm) underwent multiple dewaxing and hydration steps, and were blocked with 1% bovine serum albumin and then incubated with primary antibodies overnight at 4 °C. After washing with PBS for three times, the sections on the slides were incubated with secondary antibodies at room temperature for 2 h. Finally, the slides were mounted with DAPI-containing mounting medium (ab104139, Abcam, UK) and incubated for 5 minutes at room temperature to stain nuclei. Images were taken at random fields under a microscope.

### BALF collection

BALF samples were collected as previously described (35). In brief, an incision was made in the trachea, and the lung was lavaged twice with 0.8 mL of PBS. BALF samples were centrifuged at 300 × g for 10 minutes at 4°C to isolate the cell pellet for further analysis. Total leukocyte counts were determined using a hemocytometer.

### Flow cytometry

Flow Cytometry Cells from BALF were divided into two distinct groups (5 × 10^5^ cells/group) and subsequently stained with 10 μL of anti-F4/80-FITC (0.1 mg/mL), anti-iNOS-PE (0.2 mg/mL), or anti-CD206-PE (1:10) antibodies in 100 μL PBS for 30 minutes on ice. The analysis of the stained cells was conducted on a BD LSRII-green flow cytometer utilizing BD FACSDiva software. Data interpretation was performed with FlowJo software. Cells were first gated on forward/side scatter to exclude debris (**Supplementary Figure S2**), then on F4/80^+^ cells to identify macrophages. M1 cells were defined as F4/80^+^iNOS^+^, and M2 cells as F4/80^+^CD206^+^.

### Enzyme-linked immunosorbent assay

BALF was collected to detect the levels of albumin and LDH using a Mouse Albumin (Alb)ELISA Kit (CSB-E13878m, Cusabio, China) and a Mouse Lactate Dehydrogenase (LDH) ELISA Kit (JL13877, Jonlnbio, China) according to the manufacturer’s instruction. Absorbance was measured at 450 nm using an ELx-800 Universal Microplate Reader (BIO-TEK, Vermont, USA).

### RNA extraction and quantitative real-time PCR

RNA extraction was performed using TRIzol Reagent (Invitrogen, USA) in accordance with the manufacturer’s guidelines. cDNA synthesis was carried out using 1 μg of total RNA with the PrimeScript™ RT Reagent Kit (Takara, Japan). Real-time PCR assays were performed in 20 μL reaction volumes containing 10 μL SYBR Green Universal Master Mix (Applied Biosystems, USA), 0.5 μM forward and reverse primers (final concentration; stock primer concentration: 10 μM), and 2 μL cDNA template. Reactions were run on a Step One Sequence Detection System (Applied Biosystems, USA) under the following cycling conditions: initial denaturation at 95°C for 5 min; 40 cycles of 95°C for 15 s, 60°C for 30 s, and 72°C for 30 s; followed by a melt curve analysis from 60°C to 95°C with 0.5°C increments. All samples were analyzed in technical triplicates, and biological replicates (n = 3–6 per group) were included to ensure statistical robustness. The relative gene abundance was calculated using the 2^−ΔΔCT^ method, with GAPDH as the internal control. Primer sequences are listed **Table 1**.

**Table 1:** Primer sequence for RT-qPCR.

Gene	Primer sequence (5’-3’)
GAPDH	Forward AATGACCCCTTCATTGAC Reverse TCCACGACGTACTCAGCGC
RSV	Forward AATCGAGCCAGAAGAGAACTA Reverse GCCTTGTTTGTGGATAGTAGAG
iNOS	Forward TGGCCACCTTGTTCAGCTACG Reverse GCCAGGCCAACACAGCATAC
IL-1β	Forward TGCCACCTTTTGACAGTGATG Reverse AAGGTCCACGGGAAAGACAC
IL-6	Forward CCCCAATTTCCAATGCTCTCC Reverse CGCACTAGGTTTGCCGAGTA
TNF-α	Forward ACCCTCACACTCACAAACCA Reverse ATAGCAAATCGGCTGACGGT
Arg-1	Forward TGTCCCTATGACAGCTCCTT Reverse GCATCCACCCAATGACACAT
CD206	Forward AACCAGTTCCTTGAGCTCGG Reverse CTGATTAGGGCAGCCGGTAG
IL-10	Forward TGTCTACTCACCAAAGCGCA Reverse CCTGTGCTCATAGGCTGTC
TGF-β	Forward GAGCCGGGACGGTTCTG Reverse CAGGAACATGAGGACTCGGC

### *In vitro* studies

Primary alveolar macrophages were obtained from BALF and cultured in DMEM (4.5 g/L of glucose, 110 mg of sodium pyruvate), and 10% fetal bovine serum (FBS) using flat 96-well plates, with a cell density of 10^5^ cells per well. Cells were cultured at 37°C with 95% humidity and 5% CO_2_ for 3–4 hours to allow adherence, followed by infection and treatment under the same conditions. Subsequently, the medium was changed, and the cells were infected with RSV at a MOI set to 1. Afterward, CLS treatment (consisting of 50 μM cordycepin, 100 μg/mL of lactoferrin, and 100 μg/mL of *sargassum fusiforme* polysaccharides) or cordycepin alone (CD, 50 μM cordycepin), was applied. The cultures were then incubated for a duration of 24 h before proceeding with direct cell lysis for RNA extraction or flow cytometry analysis. The concentration of cordycepin, lactoferrin and *sargassum fusiforme* polysaccharides was referred to previous studies (14, 32, 36).

### Proliferation assay

AM cell proliferation was measured using a CCK-8 cell proliferation kit (Beyotime, China) and an EdU Immunofluorescent Assay kit (Invitrogen, USA), according to the manufacturer’s instructions. EdU solution was added to the medium for the last 4 h of the 24-h incubation period.

### Apoptosis assay

To assess apoptosis, an Annexin V-FITC/PI Apoptosis kit (E-CK-A211, Elabscience, China) was employed on a flow cytometer following the manufacturer’s instructions. TUNEL assay was carried out on paraffin sections (4 μm) using a TUNEL Assay Kit (servicebio, China) according to the manufacturer’s instruction.

### Statistical analysis

All data are expressed as the mean ± SD. All comparisons were performed using GraphPad Prism 8.2 and performed by one-way ANOVA followed by Dunnett’s tests (*in vivo* analysis) or Tukey’s tests (*in vitro* analysis) for multiple comparisons. Statistical significance was considered at p<0.05.

## Results

### CLS treatment protects against RSV-induced lung pathology in mice

**Figure 1:**
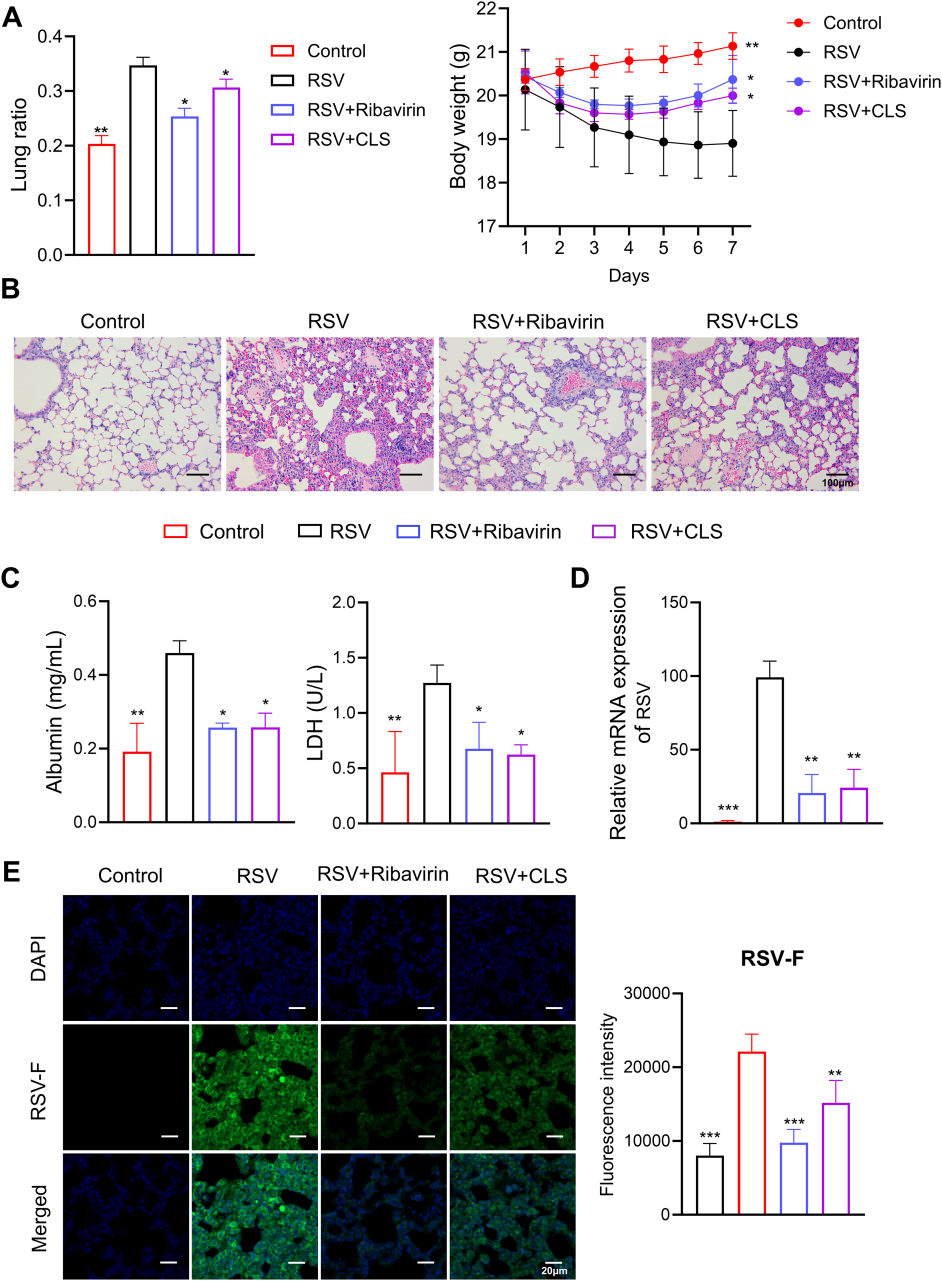
CLS treatment reduces RSV-induced lung pathology. **(A)** The lung ratio and body weight of mice. The lung ratio is presented as a percentage of lung weight relative to body weight. **(B)** Representative images of H&E-stained lung tissue sections illustrate histological differences. Scale bar: 100 µm. **(C)** Levels of albumin and LDH in BALF were quantified. **(D)** RSV mRNA expression in lung tissue was measured. **(E)** Immunofluorescence staining of RSV-F in lung tissue (green), with DAPI-stained nuclei (blue). Scale bar: 20 µm. Data are presented as mean ± SD; n=3 per group. *P<0.05, **P<0.01, ***P<0.001 compared to the RSV group (one-way ANOVA with Dunnett’s *post hoc*).

To evaluate the therapeutic potential of CLS (Cordycepin, Lactoferrin, and *Sargassum fusiforme* polysaccharides) in RSV infection, we administered CLS daily to RSV-infected mice and compared its effects with ribavirin as a positive control. The lung ratio, a measure of lung weight relative to body weight, was significantly reduced in the CLS-treated group compared to the RSV group; whereas the body weights partially recovered from reduction induced by RSV infection in the CLS group (**Figure 1A**). Histological analysis further revealed that CLS alleviated lung inflammation, as evidenced by reduced inflammatory cell infiltration and tissue damage in H&E-stained sections (**Figure 1B**). Moreover, CLS significantly decreased levels of albumin and LDH in bronchoalveolar lavage fluid (BALF), markers of lung injury (**Figure 1C**). CLS also reduced viral load, as shown by decreased RSV mRNA expression and reduced RSV-F protein levels in lung tissues. (**Figures 1D, E**). Together, these findings demonstrate the protective effects of CLS against RSV-induced lung pathology.

To assess the safety of CLS, we treated healthy mice with CLS and monitored serum levels of ALT, AST, and Scr, along with histological changes in major organs. No significant alterations were observed, indicating that CLS treatment does not adversely affect liver or kidney function or tissue integrity (**Supplementary Figure S1**).

### Protective effects of CLS Are impaired in absence of alveolar macrophages

**Figure 2:**
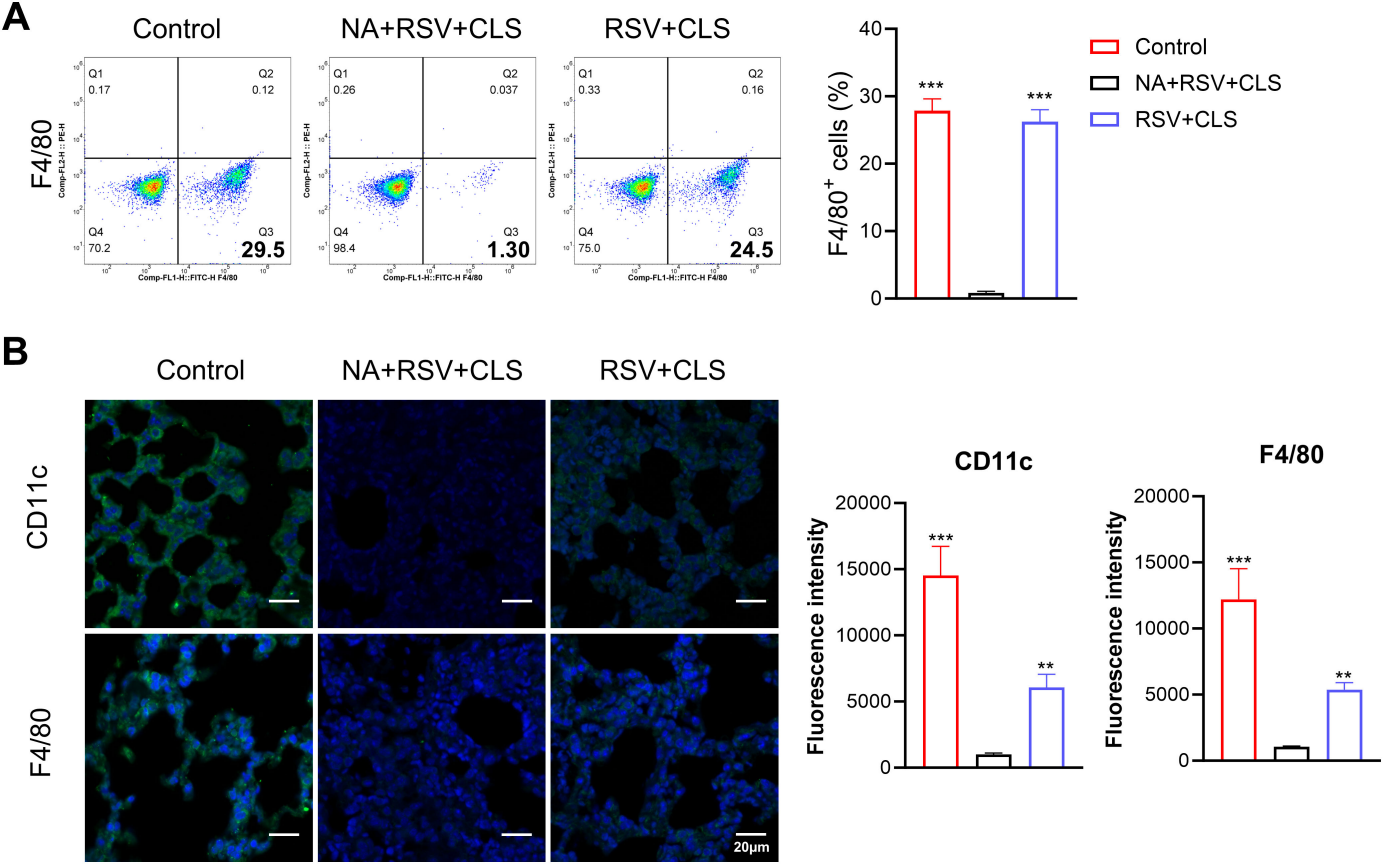
Depletion of alveolar macrophages using clodronate liposomes. **(A)** Flow cytometry analysis of F4/80^+^ cells in BALF confirms depletion. **(B)** Immunofluorescence staining of CD11c and F4/80 (green), with DAPI-stained nuclei (blue). Scale bar: 20 µm. n=3 per group. **P<0.01, ***P<0.001 compared to the NA+RSV+CLS group (one-way ANOVA with Dunnett’s *post hoc*).

To investigate the role of alveolar macrophages in the therapeutic effects of CLS, we depleted these cells using clodronate liposomes in RSV-infected mice (the NA group). Depletion efficacy was confirmed by the near absence of F4/80^+^ cells in BALF, as shown by flow cytometry (**Figure 2A**) and immunofluorescent staining (**Figure 2B**).

**Figure 3:**
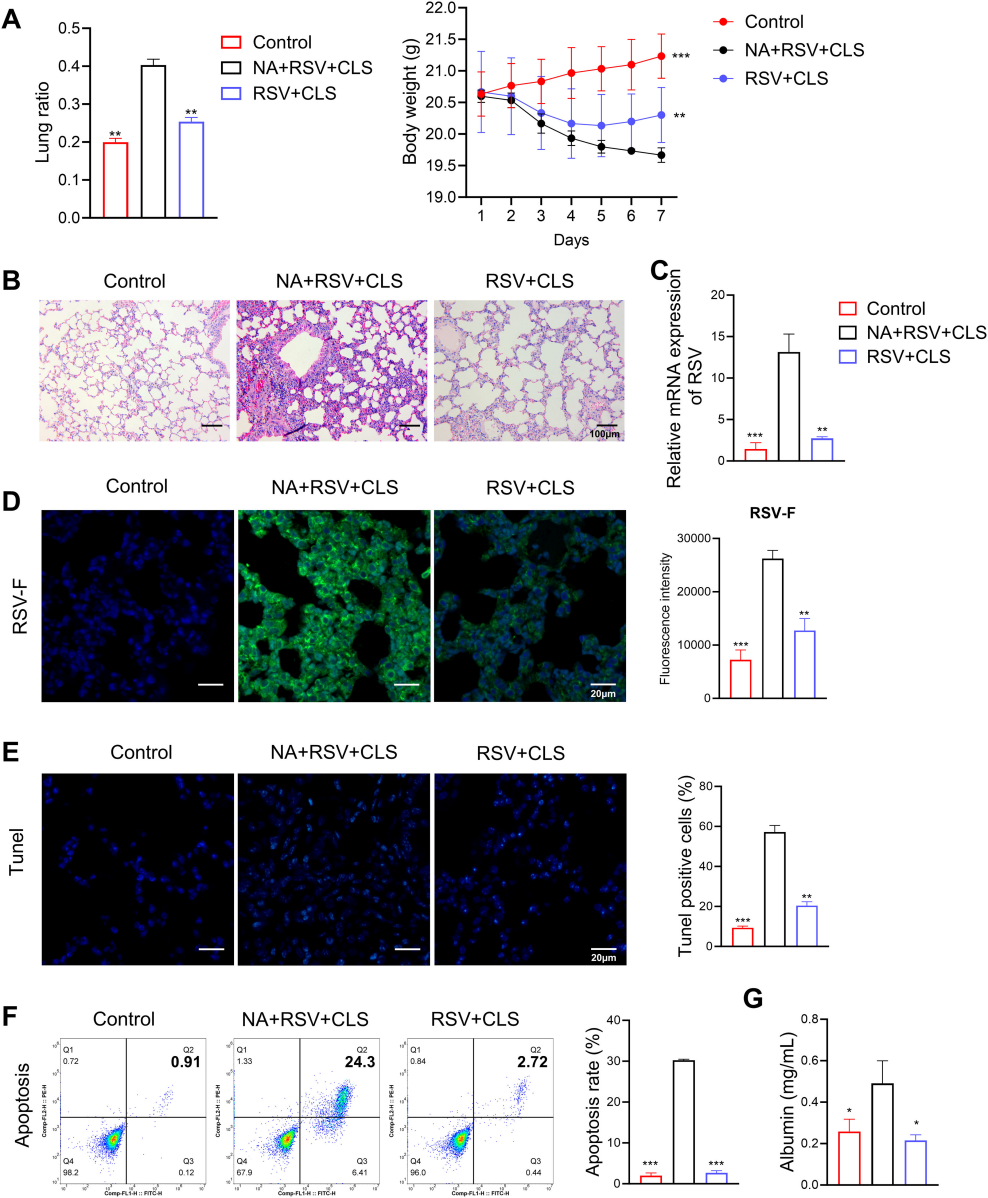
Absence of alveolar macrophages impairs CLS protection against RSV. **(A)** The lung ratio and body weight of mice. **(B)** Representative images of H&E-stained lung tissue highlight pulmonary inflammation. Scale bar: 100 µm. **(C)** Relative RSV mRNA levels in lung tissue. **(D)** Immunofluorescence staining of RSV-F (green), with DAPI-stained nuclei (blue). Scale bar: 20 µm. **(E)** Immunofluorescence staining of TUNEL, with DAPI-stained nuclei (blue). Scale bar: 20 µm. **(F)** Flow cytometry analysis of apoptosis in BALF. **(G)** Levels of albumin in BALF are shown. Data are expressed as mean ± SD; n=3 per group. *P<0.05, **P<0.01, ***P<0.001 compared to the NA+RSV+CLS group (one-way ANOVA with Dunnett’s *post hoc*).

In macrophage-depleted mice (the NA group), CLS failed to attenuate lung injury, as evidenced by persistent increased lung ratios, weight loss (**Figure 3A**), persistent pulmonary inflammation (**Figure 3B**), and elevated RSV viral load shown with RSV mRNA levels (**Figure 3C**) and stained RSV-F expression in lung tissues (**Figure 3D**). Apoptosis was significantly increased in these mice, as shown by TUNEL assays and flow cytometry (**Figures 3E, F**), and albumin levels in BALF remained elevated (**Figure 3G**). These findings suggest that alveolar macrophages are essential mediators of the protective effects of CLS.

### CLS treatment promotes M2-like polarization of macrophages in RSV-infected mice

**Figure 4:**
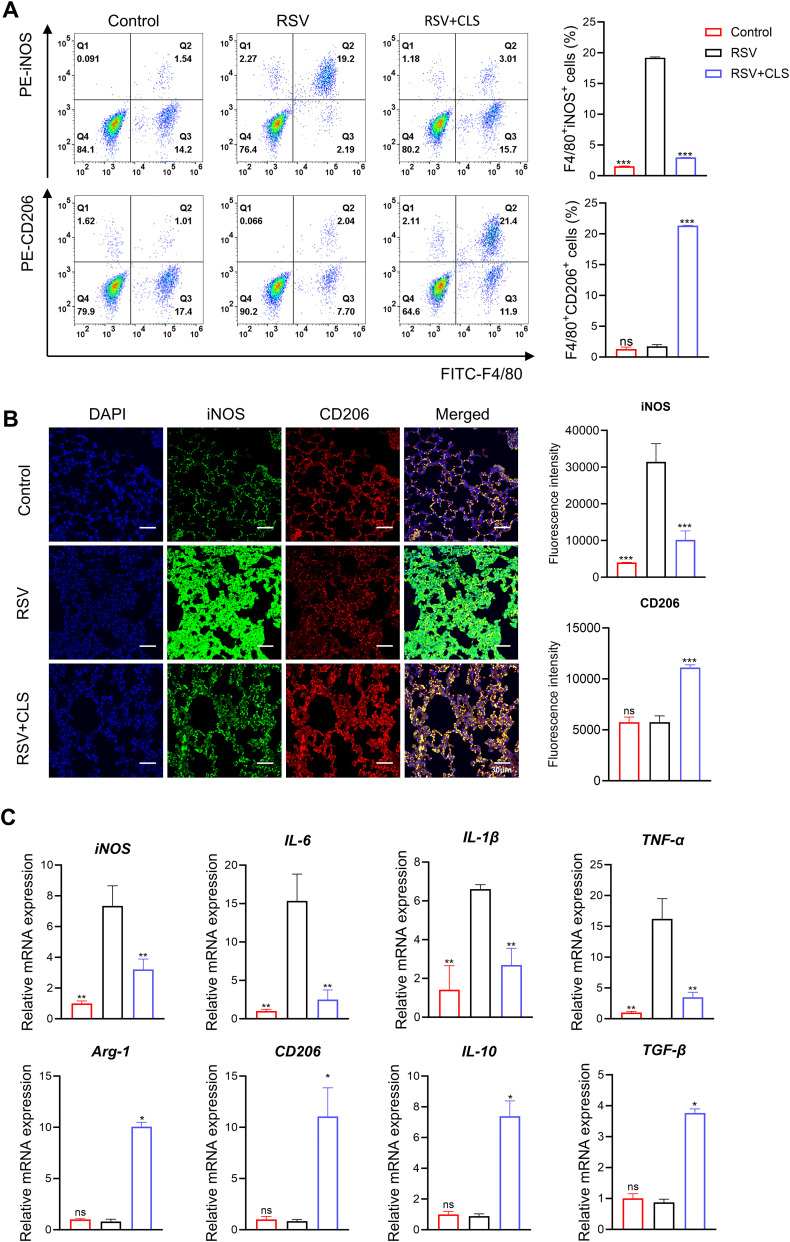
CLS promotes M2-like macrophage polarization in RSV-infected mice. **(A)** Flow cytometry analysis of BALF cells for F4/80^+^iNOS^+^ M1 cells and F4/80^+^CD206^+^ M2 cells. **(B)** Immunofluorescence staining of iNOS (green) and CD206 (red) in lung sections with DAPI nuclei (blue). Scale bar: 30 µm. **(C)** Relative mRNA expression of M1 marker (iNOS, IL-1β, IL-6, and TNF-α) and M2 marker (Arg-1, CD206, IL-10, and TGF-β). Data are shown as mean ± SD; n=3 per group. *P<0.05, **P<0.01, ***P<0.001, ns *P*>0.05 compared to the RSV group (one-way ANOVA with Dunnett’s *post hoc*).

We further explored the mechanism by which CLS exerts its effects through macrophages. Flow cytometry analysis showed that RSV infection increased the proportion of F4/80^+^iNOS^+^ M1-like macrophages. However, CLS treatment significantly reduced this population and increased the proportion of F4/80^+^CD206^+^ M2-like macrophages (**Figure 4A**). Immunofluorescence confirmed these changes, with reduced iNOS and increased CD206 expression in CLS-treated mice compared to RSV-infected mice (**Figure 4B**). RT-qPCR analysis revealed a decrease in M1 marker mRNA levels (iNOS, IL-1β, IL-6, and TNF-α) and an increase in M2 marker mRNA levels (Arg-1, CD206, IL-10, and TGF-β) in the CLS-treated group (**Figure 4C**). These results indicate that CLS promotes a shift toward M2-like macrophage polarization, contributing to its therapeutic effects.

### CLS synergy enhances RSV inhibition and macrophage survival beyond cordycepin monotherapy by amplifying M2 polarization

**Figure 5:**
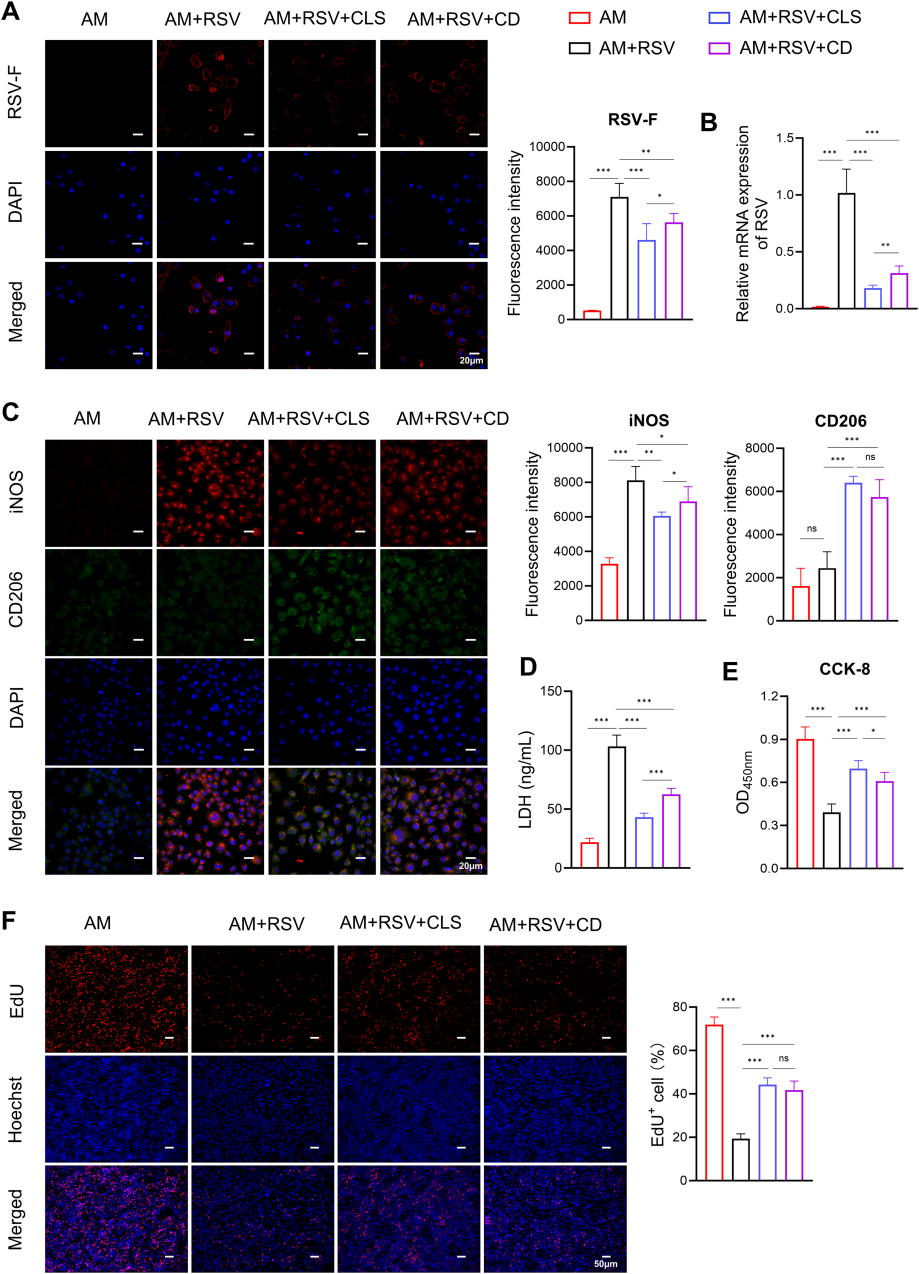
CLS combination synergistically reduces RSV infection and enhances macrophage survival compared to cordycepin monotherapy in RSV-infected cells. **(A)** Immunofluorescence staining of RSV-F (red, with blue DAPI nuclei) in alveolar macrophages (AMs) across experimental groups: ​AM controls, ​RSV-infected, ​RSV+CLS, and ​RSV+Cordycepin (CD)​. Scale bar: 20 µm. **(B)** RSV mRNA expression levels. **(C)** Dual immunofluorescence for M1 marker iNOS (red) and M2 marker CD206 (green) with DAPI nuclei (blue). Scale bar: 20 µm. **(D)** Levels of LDH in culture supernatants indicate cytotoxicity. **(E)** CCK-8 analysis of macrophage proliferation. **(F)** EdU staining of macrophages. Scale bar: 200 µm. Data are presented as mean ± SD; n=6 per group. *P<0.05, **P<0.01, ***P<0.001, ns *P*>0.05 (one-way ANOVA with Tukey’s *post hoc*).

*In vitro* experiments confirmed the role of CLS in altering macrophage polarization and reducing apoptosis. Immunofluorescence showed reduced RSV-F protein expression in both CLS-treated and cordycepin (CD)-treated alveolar macrophages compared to RSV-infected controls (**Figure 5A**). However, the CLS combination exhibited superior antiviral efficacy, lowering RSV-F expression by 40% (vs. 25% for CD alone, *P*<0.05). Similarly, RSV mRNA levels were suppressed more effectively by CLS (5.6-fold reduction) than by CD monotherapy (3.2-fold, *P*<0.01; **Figure 5B**).

RSV infection induced pronounced M1-like polarization, as evidenced by elevated iNOS staining (**Figure 5C**). While CD alone partially mitigated this shift (iNOS^+^ cells: 75% compared to RSV controls, *P*<0.05), CLS treatment further reduced iNOS^+^ cells to 57% (*P*<0.05 vs. CD group). Conversely, M2-like polarization (CD206^+^) trended higher in the CLS group (28%) compared to CD (21%), though this difference did not reach statistical significance (**Figure 5C**).

CLS also outperformed CD in reducing RSV-associated cytotoxicity. LDH levels in CLS-treated supernatants were 58% lower than in RSV controls (*P*<0.001), whereas CD alone achieved only a 39% reduction (*P*<0.001 vs. RSV; **Figure 5D**). Similarly, CCK-8 assays demonstrated that CLS restored macrophage viability to 77% of AM controls, exceeding CD monotherapy (67%, *P*<0.05; **Figure 5E**). However, EdU staining revealed no significant difference in proliferative capacity between CLS- and CD-treated macrophages (**Figure 5F**).

**Figure 6:**
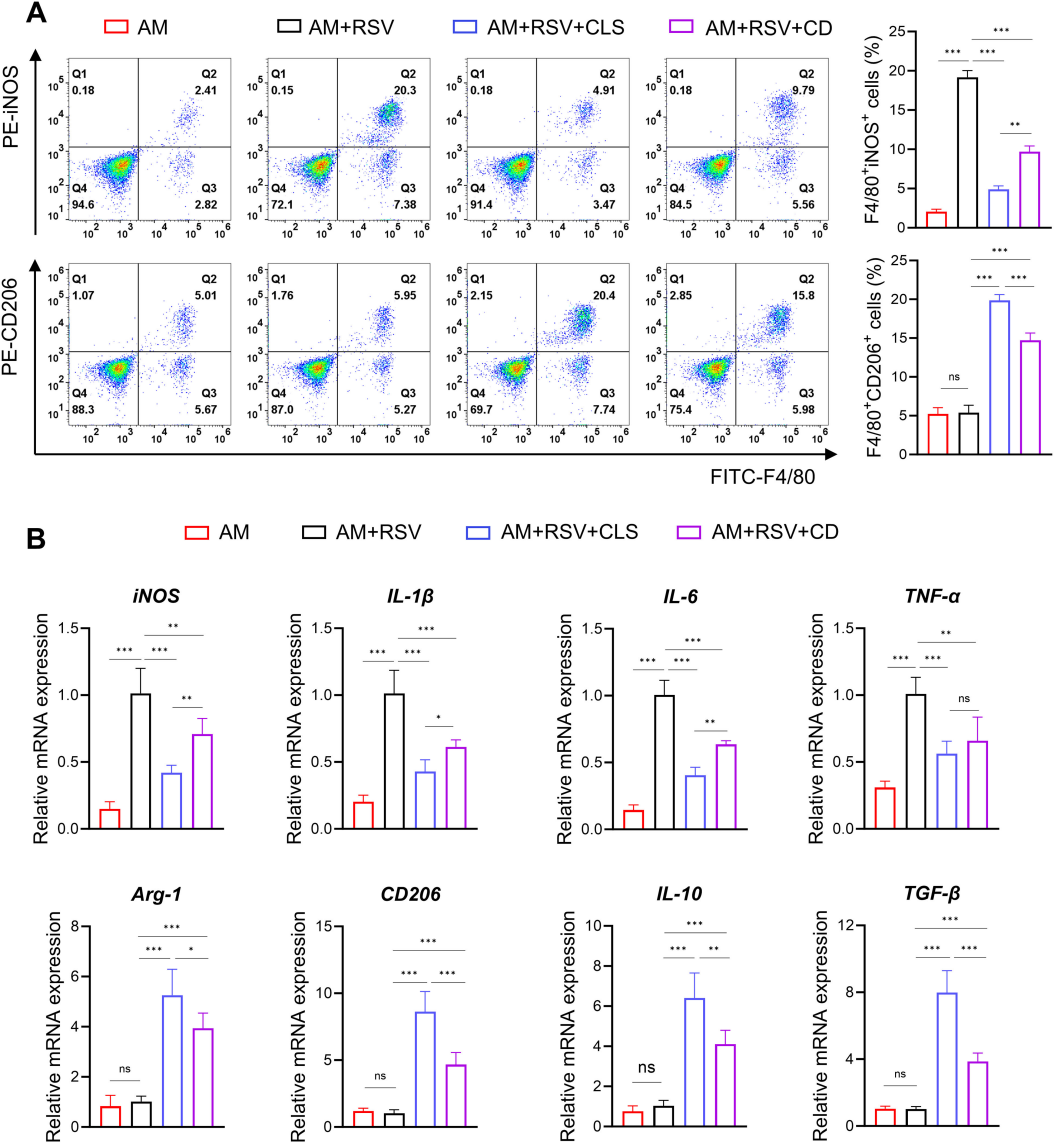
CLS synergy amplifies M2-like polarization in RSV-infected alveolar macrophages beyond cordycepin alone. **(A)** Flow cytometry analysis of F4/80^+^iNOS^+^ M1 cells and F4/80^+^CD206^+^ M2 cells. **(B)** RT-qPCR results of M1 marker mRNA (iNOS, IL-1β, IL-6, and TNF-α) and M2 marker mRNA (Arg-1, CD206, IL-10, and TGF-β) expression. Data are shown as mean ± SD; n=6 per group. *P<0.05, **P<0.01, ***P<0.001, ns *P*>0.05 (one-way ANOVA with Tukey’s *post hoc*).

Flow cytometry further delineated polarization dynamics: CLS-treated macrophages exhibited a ​2.0-fold decrease​ in F4/80^+^iNOS^+^ M1 cells compared to CD (*P*<0.01), alongside a ​1.3-fold increase​ in F4/80^+^CD206^+^ M2 cells (*P*<0.001; **Figure 6A**). mRNA analysis corroborated these trends, with CLS downregulating M1 markers (iNOS, IL-1β, IL-6, and TNF-α) by 1.1- to 1.7-fold (*P*<0.05 vs. CD) and upregulating M2 markers (Arg-1, CD206, IL-10, and TGF-β) by 1.3- to 2.0-fold (*P*<0.05; **Figure 6B**).

These results demonstrate that while cordycepin contributes to antiviral and immunomodulatory activity, the CLS combination synergistically enhances RSV Inhibition by amplifying M2-like polarization and suppressing cytotoxicity beyond monotherapy effects.

## Discussion

The present study demonstrates that the co-administration of cordycepin, lactoferrin, and *Sargassum fusiforme* polysaccharides (CLS) significantly protects against RSV-induced lung pathology in a murine model. Our findings indicate that CLS not only reduces lung injury and inflammation but also decreases viral load and promotes M2-like macrophage polarization. These results highlight CLS as a promising therapeutic candidate for RSV infections and provide novel insights into its immunomodulatory mechanisms.

The protective effects of CLS align with previous studies demonstrating the antiviral and immunomodulatory properties of its individual components (27, 37–39). Cordycepin has shown potent antiviral activity against viruses such as SARS-CoV-2 and dengue virus, primarily through mechanisms that inhibit viral replication and modulate immune responses (40–42). Similarly, lactoferrin has been reported to exhibit *in vitro* antiviral effects against RSV, although its efficacy *in vivo* remains limited (17, 18). We assumed that lactoferrin may exert as a drug enhancer as it has been reported to has synergistical effect as an antifungal adjuvant (43). *Sargassum fusiforme* polysaccharides have been recognized for their immune-enhancing and anti-inflammatory properties, including the ability to regulate macrophage activity (19, 27). A recent study reported the anti-RSV activity of *sargassum fusiforme* polysaccharides (36). Our study is among the first to investigate the synergistic effects of these compounds in the context of RSV infection, revealing that their combination amplifies therapeutic benefits by targeting both viral and immune-mediated components of disease pathogenesis.

A critical finding of this study is the promotion of M2-like macrophage polarization by CLS, as evidenced by increased expression of M2 markers (Arg-1, CD206, IL-10, and TGF-β) and decreased expression of M1 markers (iNOS, IL-1β, IL-6, and TNF-α). Previous research has shown that macrophage polarization plays a central role in determining the outcome of RSV infections, marked by excessive M1 polarization, driving hyperinflammation, epithelial damage, and viral persistence (25, 44, 45). Pro-inflammatory M1 macrophages contribute to lung injury during acute infection, while anti-inflammatory M2 macrophages are essential for tissue repair and resolution of inflammation (46–48). By promoting M2 polarization, CLS reduces pro-inflammatory cytokines like IL-1β and TNF-α, while enhancing anti-inflammatory mediators like IL-10 and TGF-β. This balance mitigates immunopathology without compromising viral clearance, as evidenced by CLS’s superior suppression of RSV-F protein. M2 macrophages further enhance viral clearance through phagocytosis of infected cells and extracellular vesicles, synergizing with cordycepin’s direct inhibition of viral RNA polymerase. Additionally, CLS’s reduction of cytotoxicity (reduction of LDH levels) and restoration of macrophage viability suggest protection against RSV-induced apoptosis, a key factor in pediatric airway hyperresponsiveness. By promoting regulatory T-cell (Treg) activation, CLS may also foster long-term immune memory, reducing susceptibility to reinfection—an essential consideration for high-risk populations.

The comparative analysis of CLS combination therapy versus cordycepin (CD) monotherapy reveals critical insights into the synergistic mechanisms underpinning RSV inhibition and macrophage polarization. While CD alone demonstrated significant antiviral activity and partial modulation of M1/M2 polarization, the CLS formulation amplified these effects substantially. Furthermore, the 1.7-fold increase in F4/80^+^CD206^+^ M2 macrophages (**Figure 6A**) and coordinated downregulation of pro-inflammatory cytokines (IL-1β, TNF-α) suggest that CLS components act on complementary pathways—lactoferrin via iron chelation to disrupt viral membranes and polysaccharides via heparan sulfate mimicry to block RSV attachment. Notably, the absence of a proliferative advantage in EdU assays (**Figure 5F**) implies that CLS synergy arises primarily from immunomodulation rather than cellular regeneration.

CLS’s therapeutic efficacy stems from its dual targeting of viral replication and host immunity. While cordycepin directly inhibits RSV polymerase, lactoferrin and *Sargassum fusiforme* polysaccharides drive immunomodulation via STAT6 and TLR4 pathways (29, 41, 49, 50). STAT6 activation, indicated by upregulated Arg-1 and CD206, promotes M2 skewing, while TLR4 engagement by polysaccharides may amplify antiviral interferon responses. This crosstalk resolves the cytokine storm while enhancing phagocytic clearance. This dual action may prevent long-term lung damage, particularly in infants at risk of post-infection bronchiolitis or asthma-like sequelae.

Our results underscore the therapeutic potential of CLS as an alternative or adjunctive treatment for RSV infections. Although newly licensed RSV vaccines (such as subunit and mRNA-based platforms) have shown efficacy in preventing severe disease, their utility is restricted to prophylactic use. There remains an urgent need for therapeutics that mitigate active infections and reduce immunopathology in vulnerable populations. Unlike current antiviral therapies, such as ribavirin, which primarily target viral replication and are associated with variable efficacy and significant side effects, CLS offers a broader mechanism of action (51, 52). By modulating the host immune response and promoting tissue repair, CLS addresses key aspects of RSV-induced pathology that are not effectively targeted by existing treatments. This immunomodulatory approach may be particularly beneficial for high-risk populations, such as infants and immunocompromised individuals, where immune dysregulation exacerbates disease severity (53). Our findings position CLS as a potential adjunctive therapy to complement vaccination strategies, particularly in cases of breakthrough infections or for individuals ineligible for vaccination.

While our study provides compelling evidence for the protective effects of CLS, several limitations must be acknowledged. First, the experiments were conducted in a murine model, which, although widely used, dose not fully replicate the immune responses to RSV in humans. The translation of these findings to clinical practice will require validation in human trials. Second, although we demonstrated that CLS promotes M2-like macrophage polarization, the precise molecular pathways underlying this effect remain unclear. Future studies should focus on identifying the signaling pathways involved and determining whether CLS directly modulates macrophage polarization or acts through intermediary immune cells. Additionally, the long-term safety and efficacy of CLS, particularly in vulnerable populations, require further investigation.

In summary, this study demonstrates the protective effects of CLS against RSV-induced lung pathology, highlighting its dual mechanism of action in reducing viral load and modulating macrophage polarization. These findings position CLS as a promising candidate for the treatment of RSV infections and provide a foundation for further research into its clinical application. By addressing both viral and immune-mediated aspects of RSV pathogenesis, CLS represents a novel and potentially transformative approach to combating this pervasive respiratory pathogen.

## Data Availability

The original contributions presented in the study are included in the article/**Supplementary Material**. Further inquiries can be directed to the corresponding author.
