# Supplementary material for: Cordycepin, lactoferrin, and Sargassum fusiforme polysaccharides protects against RSV via M2-like macrophage polarization
Source: Front Immunol. 2025 Jun 16;16:1576069. doi: 10.3389/fimmu.2025.1576069 (PMC12206873; doi:10.3389/fimmu.2025.1576069)
Supplement: Supplementary file 1 [file SupplementaryFile1.docx]

**Supplementary materials**

**Supplementary Methods**

CLS safety animal experiment

Male 6-week-old Balb/c mice were purchased from Guangdong Animal Center (Guangzhou, China) and were all given free access to tap water and a standard diet, housed in a temperature- and humidity-controlled room with a 12:12-hour light-dark cycle. They were randomly separated into two groups: CLS treatment group and control. For CLS treatment, 100 mg/kg cordycepin, 10 mg/animal lactoferrin, 200 mg/kg sargassum fusiforme polysaccharides were administered orally per day. The control group received normal saline 0.9% at an equal volume. At the end of day 7, all mice were anesthetized. The mouse heart, liver, spleen, lung and kidney tissue were removed, fixed and paraffin embedded. Blood was withdrawn from the vena cava and centrifuged at 1000g for 10min to collect serum.

**H&E staining**

Sections (4 μm) were cut from paraffin-embedded tissues, fixed in formalin (Beyotime, Shanghai, China), and stained with hematoxylin (Beyotime, Shanghai, China) and eosin (Beyotime, Shanghai, China) following a series of dewaxing process: The slices were treated with xylene for 10 minutes and subsequently dewaxed in a series of alcohol solutions (anhydrous ethanol, 90%, and 70%) before being immersed in hematoxylin solution for 5 minutes. Excess dye was rinsed off under running water, and the slices were differentiated with 1% hydrochloric acid alcohol for 10 seconds, then rinsed again under running water. They were reverted to blue with 0.6% ammonia and rinsed once more. After reversion, the sections were immersed in eosin dye for 5 minutes, and excess dye was removed under running water. Following staining, the sections underwent gradient dehydration through a series of alcohol baths (75% for 5 minutes, 95% for 5 minutes, and anhydrous ethanol for 5 minutes), and were then immersed in xylene for 5 minutes for clearing. Finally, the sections were mounted with neutral gum and observed under an optical microscope.

**AST, Scr and ALT analysis**

Serum was collected to detect the levels of ALT, AST and Scr using kits including Mouse ALT ELISA Kit (CSB-E16539m, Cusabio, China), Mouse AST ELISA Kit (CSB-E12649m, Cusabio, China), and Mouse Creatinine (Cr) ELISA kit (HB1339-Mu, Hnybio, China) following the manufactures’ instructions.

**Statistical analysis**

All data are expressed as the mean ± SD. All comparisons were performed using GraphPad Prism (GraphPad Software Version 8.2, USA) and performed Student’s T test for comparison. Statistical significance was considered at p<0.05.


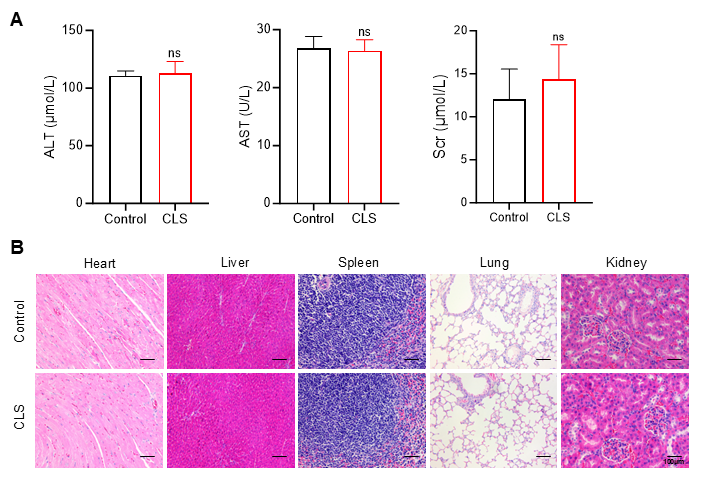


**Supplementary Figure 1.** Assessment of CLS safety in healthy mice. (A) Serum levels of ALT, AST, and Scr were measured to evaluate liver and kidney function. (B) Representative images of H&E staining in major organs, including the heart, liver, spleen, lung, and kidney. Data are expressed as mean ± SD; n=3 per group. NS indicates no significant difference (p > 0.05). Scale bar: 100 µm.


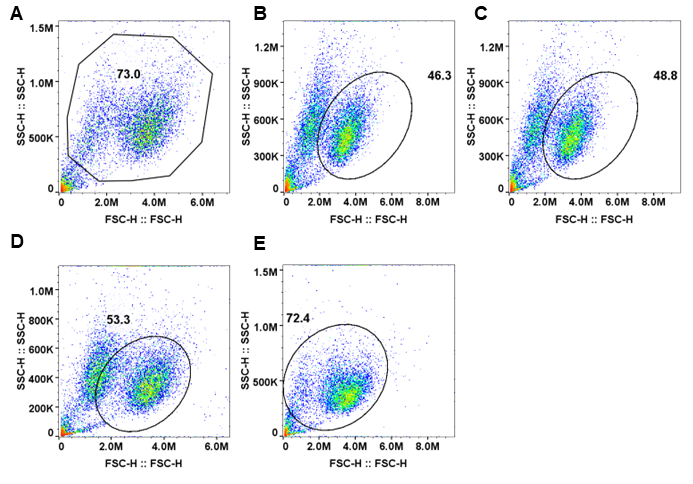


**Supplementary Figure 2.** Gating strategy for flow cytometry. (A) Gating strategy for Figure 2A to detect F4/80^+^ cells. (B, C) Gating strategy for Figure 4A to detect F4/80^+^iNOS^+^ cells (B) and F4/80^+^CD206^+^ cells (C) from BALF. (D, E) Gating strategy for Figure 6A to detect F4/80^+^iNOS^+^ cells (D) and F4/80^+^CD206^+^ cells (E) from primary alveolar macrophages.
